# Differences in Photic Entrainment of Circadian Locomotor Activity Between Lean and Obese Volcano Mice (*Neotomodon alstoni*)

**DOI:** 10.5334/jcr.145

**Published:** 2017-01-27

**Authors:** Manuel Miranda-Anaya, Dalia Luna-Moreno, Agustín Carmona-Castro, Mauricio Díaz-Muñoz

**Affiliations:** 1 Unidad Multidisciplinaria de Docencia e Investigación, Juriquilla, Qro, Facultad de Ciencias, Universidad Nacional Autónoma de México, MX; 2 CONACYT-Facultad de Ciencias Naturales, Universidad Autónoma de Querétaro, Juriquilla, Qro, MX; 3 Departamento de Biología Celular, Facultad de Ciencias, Ciudad Universitaria, Universidad Nacional Autónoma de México, Ciudad de México, MX; 4 Instituto de Neurobiología, Universidad Nacional Autónoma de México, Juriquilla, Qro, MX

**Keywords:** Circadian, Photic phase shift, Obesity, Locomotor activity, *Neotomodon alstoni*, c-Fos, VIP

## Abstract

Obesity is a growing problem worldwide with a clear impact on health status. It is also a condition that negatively affects circadian rhythms. When the mouse *Neotomodon alstoni* is fed a regular rodent chow, some individuals develop obesity, representing an opportunity to compare the effects of spontaneous obesity upon the circadian organization in this species with that observed in other rodents with induced obesity. We report differences in the free running circadian locomotor activity rhythm and in the effects of light pulses between lean and obese mice. Also, the photo-induced expression of the c-Fos protein and vasoactive intestinal peptide (VIP) in the suprachiasmatic nucleus (SCN) were examined at circadian time (CT) 14 and 22. We show that obese mice have a larger dispersion of the period of circadian locomotor rhythm in constant darkness. Photic induced phase shifts are nearly 50% shorter at CT 14, and 50% larger at CT 22 than in lean mice. The photoinduction of VIP in the SCN at CT 22 was larger in obese mice, which may be related to the differences observed in photic phase shifting. Our work indicates that the obesity in *Neotomodon* has effects on the neural mechanisms that regulate the circadian system.

## Introduction

Most living organisms have an internal clock that confers 24-h rhythmicity to their physiological processes. In natural conditions, entrainment of the clock by external periodic signals is an evolutionary advantage, because it allows rhythms to adapt and anticipate natural periodic changes [1,2,3]. Light, temperature, food availability, or even social contact can indicate the time of day; however, light transitions from dusk to dawn give the main entraining signal (*zeitgeber*) for the pacemaker in mammals, the suprachiasmatic nucleus (SCN) of the hypothalamus [4]. The major photic input to the SCN comes from the retinohypothalamic (RHT) tract [5, 6]. Under constant darkness, brief periods of light produce different effects in the phase of circadian locomotor activity. Exposure during the early subjective night causes phase delays, whereas, during the late subjective night, the exposure causes phase advances; finally, brief light exposure during the subjective day does not alter the circadian phase [7]. These behavioral changes are usually correlated with photoinduction of the protein c-Fos in the SCN [8].

Interactions among central pacemaker, peripheral oscillators, and the external environment converge in entrained output rhythms [4]. Recent studies have shown that nutritional and hormonal, food-related signals such as leptin, ghrelin, and glucose have effects on SCN physiology [9,10,11,12]; in addition, they also influence the mechanisms mediating the entrainment to light/dark cycles [13,14,15]. Therefore, they provide additional evidence that endocrine/metabolic diseases are closely related to circadian disruption [16,17,18,19,20].

Obesity has received particular attention from researchers, mainly because this condition has greatly increased worldwide and has been linked to an environment that offers an abundance of calorie-rich foods and few opportunities for physical activities [21, 22]. Diverse rodent studies relating obesity and circadian rhythms indicate different effects depending on the biological model used, particularly when entrainment is achieved in response to light pulses. For example, lipid-enriched food reduced photic phase shifting in C57 JBL/6 J mice [23], also changing fundamental properties of the pacemaker and causing the lengthening of the free-running period [24, 25]. However, *ob/ob* mice, genetically unable to produce leptin, displayed a larger photic phase shifting than wild-type mice [26], while in other rodents, such as Zucker rats, no clear differences were observed [27]. Finally, *clock* mutant mice that show altered metabolic responses [28] also show greater photic phase shifts than those observed in wild type mice [29]. Genetically obese *db/db* mice, lacking the long isoform of the leptin receptor, display longer free-running period and increased photoinduction of c-Fos during the late night than *db/+* mice do [30].

In mammals, glutamate is the main neurotransmitter involved in light transduction of the circadian response [31]; however, other peptides such as pituitary adenylate cyclase-activating polypeptide (PACAP) and vasoactive intestinal peptide (VIP) may modulate light transduction [32, 33]. For example, exogenous application of VIP resets the SCN circadian clock in a light-like manner, mediated through the VPAC2 receptor [34]. Changes in fundamental properties of circadian rhythms, such as changes in period observed in obese animals, may be related to coupling within the SCN itself as well as with peripheral oscillators [24]. An important coupling mechanism is the one mediated by VIP and its receptor VPAC2, also associated with changes in the free-running period [35]. Therefore, a significant change in VIP and/or VPAC2 receptor-associated signaling could accompany the changes where the free-running period is affected in obese mice.

Diverse animal models offer unique and powerful experimental approaches. They allow us to understand the underlying mechanisms that relate metabolic disorders to circadian disruption and to develop therapeutic strategies [17, 19, 36, 37]. It is particularly useful to understand whether the altered mechanisms in obesity occur regardless of the model used, or if they are a consequence of the specific method used to induce obesity [38]. The endemic Mexican volcano mouse, *Neotomodon alstoni,* is an advantageous model for a variety of behavioral and physiological studies, including circadian rhythms and its relation to obesity [39,40,41] and hypothalamic integration of food intake [42]. In this context, a mouse such as *N. alstoni* offers a different perspective, since some of these mice develop obesity with standard rodent diets, while others remain lean. As they grow, almost half of the population – either wild-captured or F1 offspring – becomes progressively overweight and eventually obese after 4–6 months. The body weight of these obese specimens is almost twice that of lean mice, indicating a probable biological predisposition for overweight in some sector of the population [39, 40]. In the present study, we present the differences in circadian phase shift, from lean and obese *N. alstoni* born in vivarium conditions and we address the mechanisms of such differences by studying the photoinduction of c-Fos synthesis in the SCN, as observed by others [8, 23] as well as in the presence of VIP [33, 34].

## Materials and Methods

### Animals

**Figure 1:**
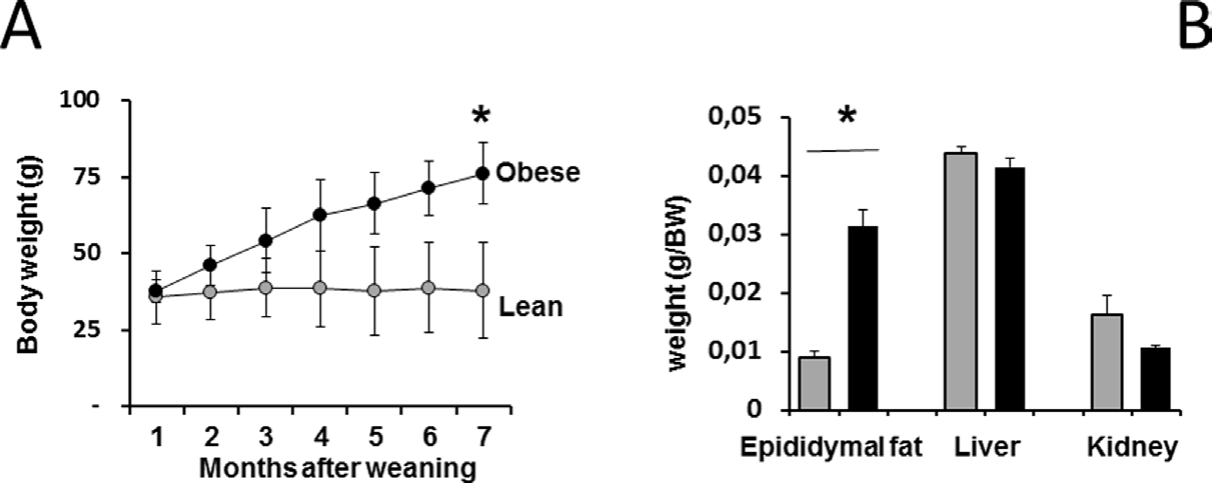
**Body weight differences between lean and obese mice.** (**A**) Body weight gain after weaning shows the difference between lean and obese used. (**B**) Epididymal fat, liver, and kidney weights are shown as grams/Body Weight (g/BW) shows a substantial change in the size of fat tissue but not in other organs.

In order to explore the effects of obesity on the circadian rhythm of locomotor activity and its response to light, 6–8 month-old male mice *Neotomodon alstoni* born and raised in captivity were used. All animals were held under a 12 h: 12 h light-dark (LD) cycle (0600–1800 h photophase, 200–250 lux). Mice were individually housed in cages containing wood chips. Food chow (Rodent Lab 5001, Purina Inc.) and tap water were provided *ad libitum*. For 6 months after weaning, body weight (BW; average ± SE) was followed in male offspring (F1), and the animals were then separated into two groups: lean (BW = 45 ± 3 g) and obese (BW = 76 ± 4 g) (Figure 1A). Mice between 50 and 60 g were not considered in this study. Obesity was characterized according to previous work [40] where obese mice were 50% heavier than lean mice and also presented elevated blood concentrations of leptin, insulin, triacylglycerides and basal glucose. In the present study, we also compared the amount of epididymal fat, and the change of size in organs such as the liver, and kidney (Figure 1B). All procedures described in this article were carried out in accordance with the ethical guidelines of the Declaration of Helsinki and National Institutes of Health Guide for the Care and Use of Laboratory Animals (NIH Publication No. 8023), institutional guidelines, and the General Law of Health for Research Studies in Mexico (NOM-062-Z00–1999). The present study was approved by the ethics committee of the Biology Department, Facultad de Ciencias, UNAM. Procedures to provide appropriate animal welfare and to reduce suffering were observed under the strict supervision of expert veterinary care.

### Methods of euthanasia

At the end of the study, mice were deeply anesthetized with sodium pentobarbital (50 mg/kg) and perfused transcardially with 50 ml of saline solution (0.9%) followed immediately by 50 ml of 4% paraformaldehyde in 0.1 M phosphate buffer (PB; pH 7.2).

### Locomotor activity recordings

Activity in a non-wheel running environment was continuously monitored using a light beam detection system and collected with data acquisition software (ACTBIO, UNAM, Mexico) as described elsewhere [40]. Each beam interruption was considered a single event. Data were collected and stored on a computer every 10 min. Each cage was placed in a light-tight wooden box equipped with a fluorescent lamp (200–250 lux), and a timer controlled LD cycles. Ventilation inside the chamber was kept constant using a small fan, and the temperature was maintained at 23–25 ºC. During LD, cage maintenance was provided once a week during the photophase, whereas when in constant darkness (DD) cleaning was performed during the subjective day under dim red light.

### Protocols. Free-running and Photic Phase-shifts

**Figure 2:**
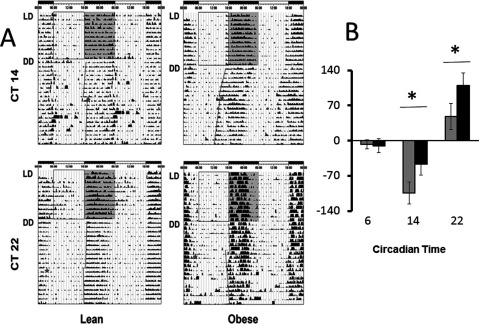
**Circadian locomotor activity and photic phase response in lean and obese mice.** In (**A**) representative actograms, star indicates the time when the light pulse (1 h) was given. Previous phase onset is projected with a solid gray line on the left side of each actogram. In (**B**) average phase shift at CT 6, 14, and 22 (n = 6, each) show differences in delays and advances between lean (white bars) and obese mice (black bars); (* p < 0.05, Student’s t test).

The phase shifts were tested at circadian times (CT: 06, 14 and 22) according to a previous work [43] considering CT12 as the time of activity onset. Phase-shift data were obtained individually by entraining the rhythm to an initial LD cycles (12: 12) for at least one week; then mice were kept in DD during 10 days before a pulse of bright white light (1 h, 250 lx) was given at different circadian times. Finally, recordings were made over 10 more days in DD (Figure 2A). Immunocytochemistry studies were performed only in mice collected at CT 14 and 22, because no differences were observed in the photic phase shift of locomotor activity at CT 6 (Figure 2B).

### Data analysis

Circadian changes of locomotor activity rhythm were analyzed in double-plotted actograms employing Actiview software (Mini Mitter, OR, USA), and significant circadian periods (p < 0.05) were obtained with the Periodogram analysis provided by CHRONOSFIT® Software [44]. Phase stability in DD was defined as the variance in the day-to-day onset of activity as indicated elsewhere [45], considering the absolute difference between the actual and projected activity onset time according to the free-running period calculated. All analyses were performed within five days of steady state rhythms for each protocol. The phase shift of the onset of activity was calculated as the difference, at the day of the light pulse, between the two estimated lines for the five days before and the five days after the photic stimulus. Comparisons between lean and obese mice at the same CT were analyzed with the nonparametric Kruskal-Wallis test and contrasted with Dunn’s Multiple Comparison test, by means of the Prism software (Graphpad®). Results were considered statistically significant when p < 0.05; all values are shown as the mean ± standard error (SE). Tail comparison in the free-running period dispersion was tested by means of an F test and paired comparisons on body weight, and Lee index were tested with the Student’s t-test.

### Immunohistochemistry (IHC)

Lean and obese mice were treated as in the phase-shifting protocol described above at CT 14 or CT 22. A control group was euthanized without receiving light pulse (–P; n = 3 each) while mice in the experimental group, were exposed to a 1-h pulse of fluorescent (200 lx) white light (+P, n = 3 each). After perfusion, brains were removed and post-fixed in paraformaldehyde solution for 24 h, then transferred successively to 10, 20, and 30% sucrose solutions in 0.1 M PB. Frozen coronal sections of 40 μm were cut with a cryostat. There are no stereotaxic studies of the brain of this rodent species; therefore, as an approximation to the medial SCN, we examined slides above the optic chiasm used as a reference. Sections were serially collected; one set of slides was processed with the c-Fos antibody (1: 1000; Santa Cruz Biotech®, CA, USA) and other series with VIP antibody (Vasoactive Intestinal Peptide Antibody; 1: 500; Immuno Star®, USA). Slides were incubated for 72 h at 4°C in PB saline (PBS), 1% of Goat Serum, and 0.3% Triton X-100 (PBSGT). Tissues were rinsed three times for 10 min in PBS and then incubated for 2 h with biotinylated secondary antibody (goat anti-rabbit; Vector Laboratories) 1: 5000 in PBSGT. Slices were rinsed three times for 10 min and incubated for 2 h with the Vectastain Elite ABC Kit (0.9% avidin and 0.9% biotin solutions; Vector® Laboratories, CA, USA) and washed three times again for 10 min in PBS. The tissue was incubated with diaminobenzidine (0.5 mg/ml, in Tris-HCl buffer 7.2) and H_2_O_2_ (35 µl, 30% H_2_O_2_). After this, sections were mounted on poly-D-lysine (SIGMA®, USA) slides and coverslipped with CC/mount (SIGMA®, USA).

### Evaluation of IHC

After IHC, slides from each experimental condition were inspected and photographed with a microscope (AXIO A1 Zeiss®, Germany) coupled to a digital camera (Axiocam Erc53), and the software Axiovision 4.8 (Zeiss®, Germany) was used for further analysis. The c-Fos immunoreactive cells in the SCN were evaluated by visually counting stained nuclei on each side of the SCN in a gridded area of about 90,000 µm^2^. VIP staining was also evaluated in each slide as the average optical density of 25 consecutive 100-µm radius fields, distributed in the SCN area, by using Image-J software (NIH, ver. 1.4). Background optical density was established in the optic chiasm and subtracted from each of the samples; average of all fields is reported as optical density.

## Results

### Body weight increase in Neotomodon F1 mice

In order to explore whether *Neotomodon* offspring had a body weight increase similar to that of the wild mice observed before [40], we followed a group of mice born in captivity until the 7^th^ month. Figure 1A illustrates the average body weight (BW) gain after weaning (n = 10 each group) for lean and obese F1 mice. Average BW (± SE) of animals at 7 months (lean = 43.3 ± 3 g; obese = 76.5 ± 2.2 g) indicated the obese mice were nearly 70% overweight (p < 0.05). Groups of lean and obese mice can be distinguished from the 3rd month after weaning in F1, and they are clearly different by the 7^th^ month. At this age, significant differences were noted in epididymal fat (~340% increase; Figure 1B, n = 18) but not in liver and kidney weight.

### Circadian rhythm in free-running and activity onset

To explore circadian parameters such as the free-running period and phase-onset stability, we analyzed the locomotor activity rhythm in free run, recorded just after entrainment to LD and before the light pulse. No statistical differences were observed in average free-running period (obese τ = 23.82 ± 0.11 h, n = 11; lean τ = 23.82 ± 0.23 h, n = 19). However, a larger dispersion of τ was observed in constant darkness in obese than in lean mice (F test for tail dispersion, p < 0.05). Day-to-day onset stability in DD in free run (absolute difference in minutes) revealed a more stable onset in lean than in obese mice (lean = 27 ± 5 min; obese = 44 ± 12 min), but the difference was not statistically significant.

### Light-induced phase shift

In order to address mechanisms of photic resetting, we tested the magnitude of phase shift at CT 06, CT 14 and CT 22. Representative examples of actograms are shown (Figure 2A). Gray squares indicate the scotophase under LD conditions; then, the circadian rhythm free runs under DD conditions. A line indicates the onset of activity used as reference, before and after a light pulse (star at either CT 14 or CT 22). In Figure 2B, the average (n = 6) of the phase shift magnitude is plotted for lean (gray bars) and obese mice (black bars). Significant differences were observed at CT 14 and CT 22: phase delays were reduced by nearly a half in obese mice at CT 14 (lean = 104 ± 22 min; obese = 47 ± 22 min; p < 0.05). However, advances were nearly 50 % larger in obese F1 than in lean mice (obese = 110 ± 25 min; lean = 48 ± 26 min; p < 0.05; Student’s t test). No differences in light sensitivity were noted at CT 6.

### c-Fos expression in SCN

**Figure 3:**
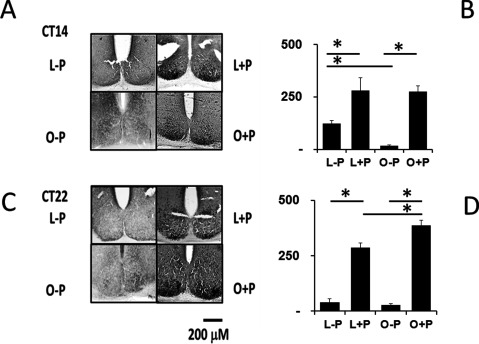
**c-Fos immunostaining in cells of the half-medial SCN.** (**A**) Representative samples. (**B**) Average (± SE) of the number of cells counted that express c-Fos. Mice without light pulse (Lean –P, Obese –P) and animals with light pulse (Lean +P, Obese +P) are shown. (* p < 0.05, Kruskal-Wallis test).

To determine if the obese *Neotomodon* mice show different photic induction of c-Fos in the SCN, we used immunohistochemistry to explore differences between obese and lean mice after a light pulse at CT 14 and CT 22. Figure 3A displays representative examples of SCN slides taken from mice without pulse (L–P/O–P), and after a light pulse (L+P/O+P) at CT 14 and CT 22. The Figure 3B presents the average number of cells positive for c-Fos (± SE, Kruskal-Wallis statistic = 152; Dunn’s Multiple Comparison test) at CT 14 and CT 22. Basal expression of c-Fos in lean mice was different between CT 14 (1258.6 ± 365.5) and CT 22 (320 ± 144; p < 0.05). After the 1-h light pulse, lean mice showed a significant increase in the cells expressing c-Fos in the SCN (lean CT 14, = 2242 ± 497; CT 22 = 2288 ± 169); in basal conditions, obese mice showed a reduced number of cells expressing c-Fos (CT 14 = 144 ± 27; CT 22 = 245 ± 53); however, c-Fos photoinduction in obese mice was not different from the observed in lean mice.

### Vip immunostaining

**Figure 4:**
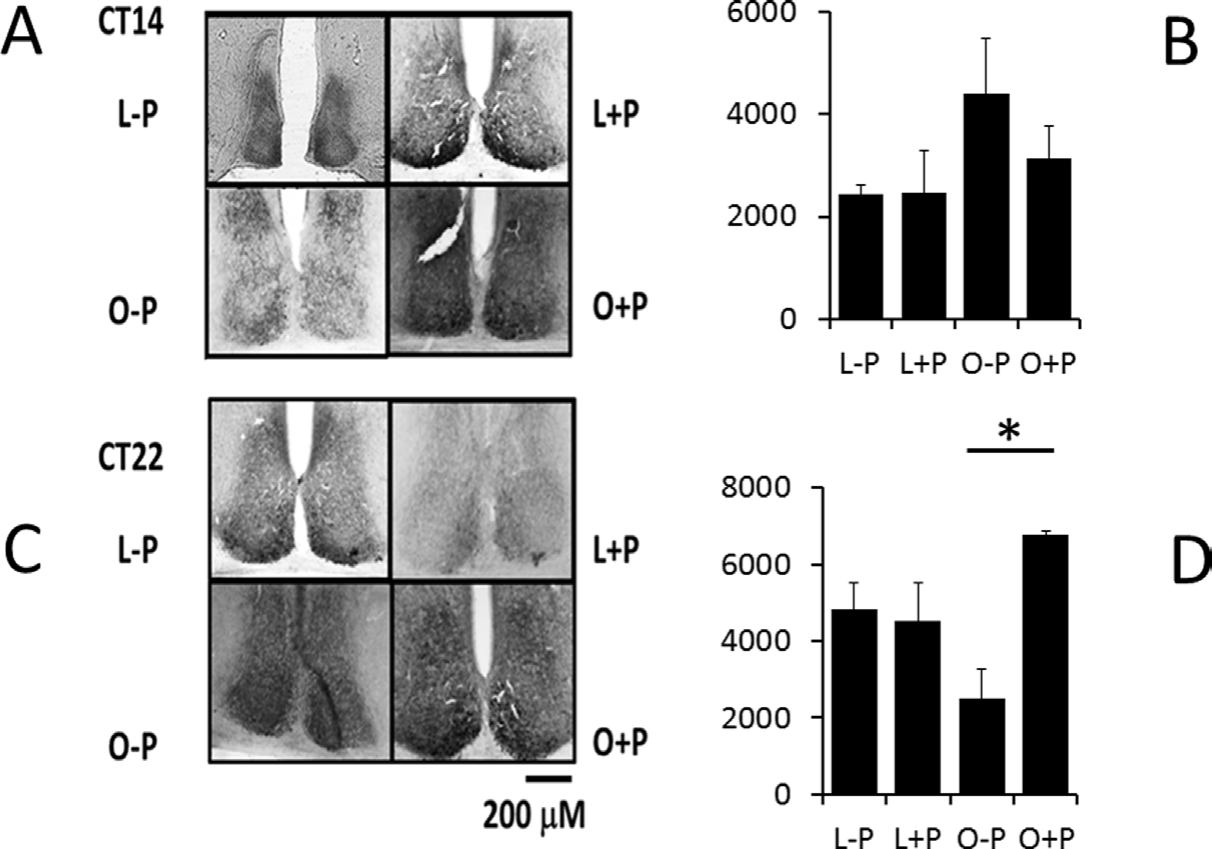
**VIP immunostaining in the half-medial SCN.** (**A**) Representative samples. (**B**) The average (± SE) of relative optic density evaluated from SCN slides in mice without light pulse (Lean –P, Obese –P) and with light pulse (Lean +P, Obese +P) are shown. (* p < 0.05, Kruskal-Wallis test).

Figure 4A shows representative slides of the SCN immunostained for VIP at CT 14 and CT 22 of lean and obese mice. In Figure 4B, the average (±SE) of VIP optical density (OD) is plotted (differences according to Kruskal-Wallis statistic, 86.51; Dunn’s Multiple Comparison test). In lean mice, basal expression of VIP (–P) was different between CT 14 (OD = 2436 ± 190) and CT 22 (OD = 4824 ± 708, Relative Units; p < 0.05); this difference was also present in obese animals, CT 14 (OD = 4401 ± 1085) and CT 22 (OD = 2515 ± 751; p < 0.05). After a light pulse, lean mice did not show significant increase in VIP (OD, CT 14 = 2456 ± 854; CT 22 = 4538 ± 997); obese mice showed a significant increase in VIP at CT 22 (OD = 6762 ± 106; p < 0.05) but no at CT 14 (OD = 3137 ± 627).

## Discussion

Metabolic dysfunction has recently been related to the circadian timing system, prompting intense interest in the linkage between these two areas of physiology research [16,17,18]. Although circadian dysfunction is associated with the development of obesity, the underlying mechanisms have not been fully established. Of particular interest in the present work was the use of free-running activity in *Neotomodon alstoni* mice, excluding any influence of wheel running on circadian rhythms [46, 47]. Vivarium conditions were the same for all animals used; some mice showed a predisposition to gain weight with regular rodent chow *ad libitum* as it occurs in wild mice (Figure 1). It is not yet known whether the metabolic handling of the caloric content of the chow diet is altered for some part of the population; nevertheless, it is notable that some individuals gain bodyweight at a different rate than others under the same environmental conditions. Obese mice presented a significant increase in epididymal fat but not in liver or kidney weight relative to body weight (Figure 1) indicating that there is no overgrow in organs but increased visceral and subdermal fat. Obese mice also show alterations that correlate with the human metabolic syndrome, such as hyperleptinemia, hyperinsulinemia, and high concentrations of circulating triacylglycerides, as well as high glycaemia and enhanced slow wave sleep [39–40].

Research in rodent models that closely mimic the changes in humans are essential [48]. Obesity in this model seems to be related to overfeeding during development, possibly due to an imbalance in the hypothalamus between orexigenic and anorexigenic endocrine signals such as leptin and ghrelin [42]. Our results indicate that obese *N. alstoni* mice show different changes in the photic response, reducing delays at CT 14 and increasing the advances at CT 22, which are different from those observed in other rodent models, such as mice fed high fat diet that present a decrease in the advance zone [23] or in the *ob/ob* mice in which phase shifts of locomotor activity are larger in the delay zone [26]. However, our results are similar to those observed in *db/db* mice at CT 22 [30]. Therefore, we cannot generalize the effect of obesity on the circadian system in all the rodent models of obesity tested, since it seems to differ among species or animal models [49]; we should not exclude the possibility that it is a species-specific response in *Neotomodon* or the way in which overweight occurs. Some individuals of *Neotomodon* mice become obese after six months of regular chow diet; therefore, obesity developed by consuming a high fat diet in few weeks may have a different impact on the circadian system than obesity achieved by regular diet.

Obese mice present hyperleptinemia and some other metabolic and neural changes related to food intake [39,40,41]. Experiments *in vitro* of the rat’s SCN indicate that leptin changes the membrane potential and the spike shape, inducing phase advances in the firing rate [9, 11]. Furthermore, leptin potentiates behavioral phase resetting by light in the advance zone of the PRC and increases *Per* transcription in mice, suggesting that leptin also exerts a positive feedback effect on the circadian pacemaker [10]. It is important to consider the potential effects of leptin during behavioral phase shifts in *Neotomodon*, since previous observations indicate high levels of leptin at late subjective night [40], which could explain an increase in the advance zone of the PRC. Ghrelin is a possible food-related entraining signal that may induce phase advance in the SCN [12]. It is possible that differences in ghrelin signaling may be present in the obesity condition in *Neotomodon* [41]. This suggestion requires experimental testing involving daily comparisons of the levels of ghrelin and its receptor between obese and lean *Neotomodon* mice, to see if there is some relationship with the differences in phase shift induced by light.

The number of cells expressing c-Fos in lean mice is different between CT 14 and CT 22 (Figure 3) because of a possible daily change in its expression, as seen in other species [50]. O-P showed a considerably smaller number of cells expressing c-Fos with no differences between CT 14 and 22, indicating that rhythmicity in c-Fos expression may also be affected in obese animals. Cells expressing c-Fos photoinduction in SCN were observed at both CT 14 and CT 22; a larger difference in some cells expressing c-Fos was noted in obese mice at CT 22 (Figure 3B). We previously found that photic phase shifts were shorter at CT 14 in obese mice ([42]; and the present work). However, we did not find an equivalent directional change in the photic induction at CT 14. It is possible that these results may be differential between the dorsomedial or ventrolateral region of the SCN, as observed in different species of rodents [50,51,52] or, it is possible that the mechanisms underlying the short delays in obese mice are affected downstream from the SCN.

At postnatal ages, a circadian rhythm in VIP expression has been observed in the SCN neurons of rats *in vivo* and *in vitro* [53–54], but in adult animals, the rhythm disappears in continuous darkness [55]. Our results suggest that basal expression of VIP in the *Neotomodon* SCN may show changes along the day (Figure 4B). Obese mice displayed a notable increasing in VIP at CT 22 after the light pulse but without significant differences at CT 14. Similar to the light effect, exogenous application of VIP resets the SCN circadian clock by acting on the VPAC2 receptor [33]. In SCN neurons, the sensitivity of this receptor to VIP depends on the time of day [56, 57], which may explain the different effects of a light pulse in lean mice at CT 14 and CT 22, and also the larger dispersion of the free-running periods [34, 58]. Moreover, the results of increasing levels of VIP could explain the increased phase advance observed in the obese mice. The possibility that a differential expression of VPAC2 receptors occurs in the SCN of obese *Neotomodon* should be tested. In a previous study, we observed differences in VPAC2 expression [42] as well as more unstable phase angle with the zeitgeber in obese *Neotomodon* [40]. In the present study, a larger dispersion of the free-running period was observed in obese animals, compared with a lengthening in period observed in obese mice fed with high-lipid diets [29]. Hence, the lability of the clock could be species-specific [7], and it may be differentially affected by a nutritional condition like obesity taking into account the way in which this condition is achieved.

Together, the results presented here suggest that the obesity condition in *Neotomodon alstoni* affects the way in which the circadian pacemaker oscillates in constant conditions and how it responds to photic stimulation. Our results contrast with other models of obese rodent tested. The advantage of studying the mechanisms affected in a species where obesity is somehow “spontaneous” is the comparison with other animal models in which obesity is induced by genetic selection or nutritional manipulations. It would also be important to consider if the changes observed are present in other rodent models where some individuals, even within the same strain, show resistance to protocols of diet-induced obesity [59,60,61]. Studying nontraditional species may improve our understanding of the links between circadian regulation and metabolic disorders. The fact that mice develop obesity spontaneously also suggests that the diet would be hyper-caloric for this species, and some animals show resistance to the development of obesity. The present paper is an original contribution to the effect of obesity upon the circadian organization in different animal models to better understand a health condition in humans.

## References

[1] Aschoff J (1960). Exogenous and endogenous components in circadian rhythms. Cold Spring Harb Symp Quant Biol.

[2] Pittendrigh CS (1960). Circadian rhythms and the circadian organization of living systems. Cold Spring Harb Symp Quant Biol.

[3] Hut RA, Beersma DGM (2011). Evolution of time-keeping mechanisms: early emergence and adaptation to photoperiod. Philos Trans R Soc LondonSeries B. Biol Sci.

[4] Menaker M, Murphy ZC, Sellix MT (2013). Central control of peripheral circadian oscillators. Curr Opin Neurobiol.

[5] Meijer JH, Schwartz WJ (2003). In search of the pathways for light-induced pacemaker resetting in the suprachiasmatic nucleus. J Biol Rhythms.

[6] Moore RY (1997). Circadian Rhythms: Basic Neurobiology and Clinical Applications. Annu Rev Med.

[7] Pittendrigh CS, Daan S (1976). A functional analysis of circadian pacemakers in nocturnal rodents. J. Comp. Physiol. A.

[8] Morin LP, Allen CN (2006). The circadian visual system, 2005. Brain Research Reviews.

[9] Inyushkin AN, Bhumbra GS, Dyball REJ (2009). Leptin modulates spike coding in the rat suprachiasmatic nucleus. J Neuroendocrinol.

[10] Mendoza J, Lopez-Lopez C, Revel FG, Jeanneau K, Delerue F, Prinssen E (2011). Dimorphic effects of leptin on the circadian and hypocretinergic systems of mice. J Neuroendocrinol.

[11] Prosser RA, Bergeron HE (2003). Leptin phase-advances the rat suprachiasmatic circadian clock in vitro. Neurosci Lett.

[12] Yannielli PC, Molyneux PC, Harrington ME, Golombek DA (2007). Ghrelin effects on the circadian system of mice. J Neurosci.

[13] Challet E, van Reeth O, Turek FW (1999). Altered circadian responses to light in streptozotocin-induced diabetic mice. Am J Physiol.

[14] Sage D, Ganem J, Guillaumond F, Laforge-Anglade G, François-Bellan A-M, Bosler O (2004). Influence of the corticosterone rhythm on photic entrainment of locomotor activity in rats. J Biol Rhythms.

[15] Bray MS, Young ME (2006). Circadian rhythms in the development of obesity: potential role for the circadian clock within the adipocyte. Obesity Rev.

[16] Green CB, Takahashi JS, Bass J (2008). The Meter of Metabolism. Cell.

[17] Bass J, Takahashi JS (2010). Circadian integration of metabolism and energetics. Science.

[18] Froy O (2010). Metabolism and circadian rhythms – Implications for obesity. Endocr Rev.

[19] Baron KG, Reid KJ (2014). Circadian misalignment and health. Int Rev Psychiatry.

[20] Golombek DA, Casiraghi LP, Agostino PV, Paladino N, Duhart JM, Plano SA (2013). The times they’re a-changing: Effects of circadian desynchronization on physiology and disease. J Physiol. Paris.

[21] Berthoud H-R, Berthoud H-R, Morrison C, Morrison C (2008). The brain, appetite, and obesity. Annu Rev Psychol.

[22] Ginsberg HN, Maccallum PR (2009). The obesity, metabolic syndrome, and type 2 diabetes mellitus pandemic: Part I. Increased cardiovascular disease risk and the importance of atherogenic dyslipidemia in persons with the metabolic syndrome and type 2 diabetes mellitus. J Cardiometab Syndr.

[23] Mendoza J, Pévet P, Challet E (2008). High-fat feeding alters the clock synchronization to light. J Physiol.

[24] Kohsaka A, Laposky AD, Ramsey KM, Estrada C, Joshu C, Kobayashi Y (2007). High-Fat Diet Disrupts Behavioral and Molecular Circadian Rhythms in Mice. Cell Metab.

[25] Hsieh MC, Yang SC, Tseng HL, Hwang LL, Chen CT, Shieh KR (2010). Abnormal expressions of circadian-clock and circadian clock-controlled genes in the livers and kidneys of long-term, high-fat-diet-treated mice. Int J Obes (Lond).

[26] Sans-Fuentes MA, Díez-Noguera A, Cambras T (2010). Light responses of the circadian system in leptin-deficient mice. Physiol Behav.

[27] Murakami DM, Horwitz BA, Fuller CA (1995). Circadian rhythms of temperature and activity in obese and lean Zucker rats.

[28] Turek FW, Joshu C, Kohsaka A, Lin E, Ivanova G, Mcdearmon E (2005). Obesity and Metabolic Syndrome in Circadian Clock Mutant Mice.

[29] Vitaterna MH, King DP, Chang AM, Kornhauser JM, Lowrey PL, McDonald JD, Dove WF, Pinto LH, Turek FW, Takahashi JS (1994). Mutagenesis and mapping of a mouse gene, Clock, essential for circadian behavior. Science.

[30] Grosbellet E, Dumont S, Schuster-Klein C, Guardiola-Lemaitre B, Pevet P, Criscuolo F (2016). Circadian phenotyping of obese and diabetic db/db mice. Biochimie.

[31] Golombek DA, Rosenstein RE (2010). Physiology of Circadian Entrainment. Physiol Rev.

[32] Kawaguchi C, Tanaka K, Isojima Y, Shintani N, Hashimoto H, Baba A (2003). Changes in light-induced phase shift of circadian rhythm in mice lacking PACAP. Biochem Biophys Res Commun.

[33] Hughes TL, Piggins HD (2008). Behavioral responses of Vipr2-/- mice to light. J Biol Rhythms.

[34] Piggins HD, Cutler DJ (2003). The roles of vasoactive intestinal polypeptide in the mammalian circadian clock. J Endocrinol.

[35] Aton SJ, Colwell CS, Harmar AJ, Waschek J, Herzog ED (2005). Vasoactive intestinal polypeptide mediates circadian rhythmicity and synchrony in mammalian clock neurons. Nat Neurosci.

[36] Arble DM, Ramsey KM, Bass J, Turek FW (2010). Circadian disruption and metabolic disease: Findings from animal models. Best Pract Res Clin Endocrinol Metab.

[37] Fonken LK, Nelson RJ (2014). The effects of light at night on circadian clocks and metabolism. Endocr Rev.

[38] Kennedy AJ, Ellacott KLJ, King VL, Hasty AH (2010). Mouse models of the metabolic syndrome. Dis Model Mech.

[39] Fuentes-Granados C, Duran P, Carmona-Castro A, Cárdenas-Vázquez R, Miranda-Anaya M (2012). Obesity alters the daily sleep homeostasis and metabolism of the volcano mouse Neotomodon alstoni. Biol Rhythm Res.

[40] Carmona-Alcocer V, Fuentes-Granados C, Carmona-Castro A, Aguilar-González I, Cárdenas-Vázquez R, Miranda-Anaya M (2012). Obesity alters circadian behavior and metabolism in sex-dependent manner in the volcano mouse Neotomodon alstoni. Physiol Behav.

[41] Fuentes-Granados C, Miranda-Anaya M, Samario-Román J, Moreno-Sáenz E, Carmona-Castro A, Cárdenas-Vázquez RJ (2010). Circadian locomotor activity and response to different light conditions in the Volcano mouse, Neotomodon alstoni (Merriam, 1898). Biol Rhythm Res.

[42] Báez-Ruiz A, Luna-Moreno D, Carmona-Castro A, Cárdenas-Vázquez R, Díaz-Muñoz M, Carmona-Alcocer V, Fuentes-Granados C, Manuel MA (2014). Hypothalamic expression of anorexigenic and orexigenic hormone receptors in obese females Neotomodon alstoni: effect of fasting. Nutr Neurosci.

[43] Miranda-Anaya M, Carmona-Alcocer V, Carmona-Castro A (2016). Effects of obesity on circadian photic entrainment of locomotor activity in wild mice Neotomodon alstoni. Biol Rhythm Res.

[44] Zuther SGB (2009). No Title [Internet]. http://www.ma.uni-heidelberg.de/inst/phar/lehre/chrono.html.

[45] Iwahana E, Karatsoreos I, Shibata S, Silver R (2008). Gonadectomy reveals sex differences in circadian rhythms and suprachiasmatic nucleus androgen receptors in mice. Horm Behav.

[46] Edgar DM, Dement WC (1991). Regularly scheduled voluntary exercise synchronizes the mouse circadian clock. Am J Physiol.

[47] Mistlberger R, Holmes M (2000). Behavioral feedback regulation of circadian rhythm phase angle in light-dark entrained mice. Am J Physiol Regul Integr Comp Physiol.

[48] Brown L, Panchal SK Rodent models for metabolic syndrome research. J Biomed Biotechnol.

[49] Lutz TA, Woods SC (2012). Overview of Animal Models of Obesity. UNIT 5.61 Current Protocols in Pharmacology. Current Protocols in Pharmacology.

[50] Guido ME, Goguen D, De Guido L, Robertson HA, Rusak B (1999). Circadian and photic regulation of immediate-early gene expression in the hamster suprachiasmatic nucleus. Neuroscience.

[51] Beaulé C, Amir S (1999). Photic entrainment, and induction of immediate-early genes within the rat circadian system. Brain Res.

[52] Beaule C, Arvanitogiannis A, Amir S (2001). Light suppresses Fos expression in the shell region of the suprachiasmatic nucleus at dusk and dawn: implications for photic entrainment of circadian rhythms. Neuroscience.

[53] Takahashi Y, Okamura H, Yanaihara N, Hamada S, Fujita S, Ibata Y (1989). Vasoactive intestinal peptide immunoreactive neurons in the rat suprachiasmatic nucleus demonstrate diurnal variation. Brain Res.

[54] Shinohara K, Honma S, Katsuno Y, Abe H, Honma K (1994). Circadian rhythms in the release of vasoactive intestinal polypeptide and arginine-vasopressin in organotypic slice culture of rat suprachiasmatic nucleus. Neurosci Lett.

[55] Ban Y, Shigeyoshi Y, Okamura H (1997). Development of vasoactive intestinal peptide mRNA rhythm in the rat suprachiasmatic nucleus. J Neurosci.

[56] Reed HE, Meyer-Spasche A, Cutler DJ, Coen CW, Piggins HD (2001). Vasoactive intestinal polypeptide (VIP) phase-shifts the rat suprachiasmatic nucleus clock in vitro. Eur J Neurosci.

[57] Dragich JM, Loh DH, Wang LM, Vosko AM, Kudo T, Nakamura TJ (2010). The role of the neuropeptides PACAP and VIP in the photic regulation of gene expression in the suprachiasmatic nucleus. Eur J Neurosci.

[58] Maywood ES, Reddy AB, Wong GKY, O’Neill JS, O’Brien JA, McMahon DG (2006). Synchronization and maintenance of timekeeping in suprachiasmatic circadian clock cells by neuropeptidergic signaling. Curr Biol.

[59] Rossmeisl M, Rim JS, Koza RA, Kozak LP (2003). Variation in type 2 diabetes-related traits in mouse strains susceptible to diet-induced obesity. Diabetes.

[60] Surwit RS, Feinglos MN, Rodin J, Sutherland A, Petro AE, Opara EC (1998). Differential effects of fat and sucrose on body composition in C57BL/6 and A/J mice. Metabolism.

[61] Prpic V, Watson PM, Frampton IC, Sabol MA, Jezek GE, Gettys TW (2002). Adaptive changes in adipocyte gene expression differ in AKR/J and SWR/J mice during diet-induced obesity. J Nutr.

